# Predicting BPD: Lessons Learned From the Airway Microbiome of Preterm Infants

**DOI:** 10.3389/fped.2019.00564

**Published:** 2020-02-04

**Authors:** Samuel J. Gentle, Charitharth Vivek Lal

**Affiliations:** Department of Pediatrics, University of Alabama at Birmingham, Birmingham, AL, United States

**Keywords:** airway microbiome, bronchopulmonary dysplasia, probiotics, lung disease, prematurity

## Abstract

Bronchopulmonary dysplasia (BPD) is the chronic lung disease of prematurity with an operational definition, various different clinical phenotypes, and a complex, multifactorial etiology. Newer unbiased systems biology approaches have identified various “omic” factors associated with the pathogenesis and prediction of BPD. Recent microbi “*omic*” studies have discovered that airways of newborns harbor a low biomass but distinct microbiome signature as early as at the time of birth. This early airway microbiome may serve to prime the host immune system and may play a role in modulating the infant's future susceptibility to severe BPD development. Temporal changes are observed in airway microbiome of preterm infants from birth to the diagnosis of BPD, with an overall decrease in bacterial diversity, and development of a relative dysbiosis marked by increased *Gammaproteobacteria* and decreased *Lactobacilli* abundance. This review will summarize previous investigations of the airway microbiome in preterm infants, appraise the utility of using the airway microbiome to predict BPD development, discuss possible molecular mechanisms involved, and speculate on future microbiome-mediated therapeutics for BPD.

## Introduction

Distinct microbial populations exist throughout the human body and have been the subject of investigation relating to human disease pathogenesis, susceptibility, and progression. The airway microbiome has been studied in the context of multiple pulmonary diseases including chronic obstructive pulmonary disease, asthma, and cystic fibrosis (1–4). Bronchopulmonary dysplasia (BPD), the most common chronic lung disease of prematurity, may result from lung injury due to a range of factors including infection, respiratory support, edema, and oxygen toxicity. Recent investigations have described differences in the airway microbiome of preterm infants that may influence susceptibility of BPD development, suggesting another parallel pathway contributory to abnormal lung development. In this manuscript, we review the potential origins of the airway microbiome, plausible covariates that influence the airway microbiome, investigations exploring associations between microbial communities and BPD susceptibility, potential mechanisms for microbiome-derived lung maldevelopment, and future microbiome-based targeted therapeutic approaches.

## Origins of the Infant Airway Microbiome

How, when, and from what source(s) does the infant's airway microbiome originate? It is unclear when the airway microbiome is first established. Study of the in utero and early postnatal microbiome suggests the possibility of a fetal microbiome preceding birth. *Ureaplasma* and *Mycoplasma* have been reported invaders of the amniotic cavity associated with intraamniotic infection and preterm labor (5, 6) and have been associated with BPD development and thus therapeutically targeted (7, 8). A distinct placental microbiome has previously been described and most closely resembles the oral microbiome (9). More recent investigations of the placental microbiome report that the placental microbiome is of low abundance and low biomass (10, 11), but a very recent report from De Goffau et al. (12) refutes this claim. According to De Goffau's study, placenta does not harbor a microbiome but may contain potential pathogens. In healthy, term pregnancies, the amniotic cavity likely remains sterile until amniotic membrane rupture (13). Bacteria have also been detected from infant meconium samples (14) and cord blood (15), further suggesting that the fetal environment may not be sterile.

Our group conducted a study in which tracheal aspirate samples from infants were collected at birth or within 6 h of birth, and each sample was found to harbor a diverse microbiome signature (16). As majority of these samples were collected right at birth, most soon after cesarean sections, we speculate that airway microbiome signature at birth could have fetal origins. Previous studies of mother–infant dyads have identified similarities between the microbiome of the amniotic fluid and infant meconium highly abundant in Proteobacteria and Firmicutes, followed by an increase in diversity and later resemblance between the breast milk and infants' intestinal microbiome (17). The placental microbiome reported by prior investigations (9), largely composed of Proteobacteria and Firmicutes, also resembles that of the airway microbiome in infants at birth that our group has reported (16). However, studies of mother–infant dyads have not concurrently investigated the amniotic fluid, placenta, and airway microbiome at birth, and the actual origin of the early airway microbiome remains unknown.

## What Factors Influence the Airway Microbiome?

Before discussion of the airway microbiome and BPD susceptibility, it is critical to consider other clinical covariates that may influence infants' airway microbiome. A common variate of investigation has been antibiotic exposure. Intrapartum antibiotics have been reported to influence the oral microbiome of infants, including a reduction in the abundance of *Lactobacillus* (18). However, given the high risk for infection in the preterm population, antibiotics are a frequent exposure making such analyses challenging. In a longitudinal study of the airway microbiome from tracheal aspirates in preterm infants, all infants were exposed to antibiotics within the first 21 postnatal days with a median duration of 12 exposure days (19). Other studies in which fewer infants were exposed to antibiotics did not report differences in bacterial diversity between exposed and unexposed infants (20). We have previously reported a similar airway microbiome at birth in infants, regardless of prenatal antibiotic exposure. Additionally, despite multiple courses of antibiotic exposure, we observed comparable dysbiotic changes among infants that developed BPD (16).

Given that cesarean delivery may preclude exposure to vaginal flora, the influence of delivery mode has been evaluated. Studies analyzing the gut microbiome have demonstrated longitudinal differences persisting for a year after birth in cesarean born infants, including a decrease in *Bacteroides* abundance (21). Our group has reported that the airway microbiome did not differ based on whether infants were born via vaginal or cesarean delivery mode (16). In another study, infants born by vaginal delivery had an airway microbiome representative of vaginal flora, while the airway microbiome of infants born via cesarean delivery resembled the skin flora (22).

Breast milk influences the maturation of the intestinal microbiome with little evidence analyzing the influence of breast milk exposure and the airway microbiome. A study of infant–mother dyads (*N* = 107) compared the microbiome of the infant stool, breast milk, and areolar skin. While these microbial communities were distinct, the infant gut microbiome more closely resembled the maternal breast milk and areolar microbiome compared to randomly chosen comparison groups (23). Whereas, the intestinal microbiome may not seem relevant to pulmonary disease, correlations between the gut microbiome and lung diseases of childhood including asthma and cystic fibrosis have previously been reported (24, 25). Interestingly, breastfed infants have more abundant *Lactobacilli* and less abundant *Proteobacteria* compared to formula-fed infants (21), a similar differentiating feature of BPD-resistant infants compared to BPD-susceptible infants (16). Additionally, a meta-analysis comparing human milk to formula milk found decreased odds of BPD development in human milk-exposed infants (OR 0.78; 95% CI 0.68–0.88); however, the quality of evidence was low (26). More evidence is needed relating human milk to the airway microbiome given the overlap in microbial composition observed in breast milk-fed infants and BPD-resistant infants.

## The Airway Microbiome at Birth

Given the possibility that bacteria may be present in the fetal environment, multiple investigators have described the airway microbiome at birth. However, characterizing the airway microbiome of infants presents several challenges including inaccessibility of the lower airway, the requirement of an invasive sampling technique (typically endotracheal intubation), and a low biomass necessitating molecular techniques susceptible to contamination. Despite these challenges, bacterial DNA have been observed in tracheal aspirates collected at the time of intubation in preterm infants (20) with changes in diversity occurring after the third postnatal day (19). From these studies, Proteobacteria (*Acinetobacter* spp.) and Firmicutes were the predominant phyla and *Staphylococcus, Ureaplasma parvum*, and *Ureaplasma urealyticum* were the most frequently identified organisms. Other observational studies of tracheal aspirates in preterm infants have found a similar predominance of *Staphylococcus* and *Ureaplasma* (27, 28). In isolates taken at birth or within 6 h of birth, we have reported a low biomass airway microbiome in both term and preterm infants. Firmicutes and Proteobacteria were predominant bacterial phyla with additional detection of Actinobacteria, Bacteroidetes, Tenericutes, Fusobacterium, Cyanobacteria, and Verrucomicrobia (16).

## Airway Microbial Signatures and BPD Development

Before the plausible contributions of the airway microbiome to BPD development are discussed, it is important to acknowledge that BPD is a not a concise disease, but rather consists of a range of clinical phenotypes resulting from heterogeneous prenatal and postnatal exposures. However, an underlying commonality to mechanisms of BPD development is pulmonary inflammation secondary to exposures such as mechanical ventilation, hyperoxia, pre- or postnatal infection, or fluid retention (29). These insults all occur to a developing, premature lung, thereby altering its developmental course. Nevertheless, differences in the airway microbiome between healthy and BPD susceptible preterm infants less likely represents an acute infection of the airway and more likely reflects respiratory dysbiosis, such as the predominance of Proteobacteria observed in disease states such as chronic obstructive pulmonary disease resulting in a dysregulated immune response (1, 2, 30).

A recent systematic review identified six studies that have evaluated the airway microbiome in preterm infants that developed BPD (31). The majority of included studies used amplification of the variable regions of 16S rRNA for bacterial sequencing (16, 19, 20, 27). In these studies, the airway microbiome was first described either at birth or in the early postnatal period and was subsequently characterized at different postnatal sampling intervals. The airway microbiome of infants developing BPD have been contrasted with different groups, including full-term matched controls and preterm infants that did not develop BPD. In comparing the airway microbiome at birth between infants that did and did not develop BPD, less bacterial diversity has been reported in infants that later developed BPD (20) and we have reported a more diverse microbiome in full-term post-menstrual age (PMA) matched infants at birth compared to infants that develop BPD (16). These differences in microbiome diversity may last into adulthood, as adult survivors with a history of BPD have a less diverse airway microbiome compared to preterm infants that did not develop BPD and healthy controls (32).

Studies analyzing microbial signatures and the outcome of BPD have found similar results but some institutional differences. We have reported that BPD susceptible infants have more abundant Proteobacteria and less abundant Firmicutes compared to PMA matched infants. These changes in microbial composition were also noted over time in infants developing BPD. Regarding individual bacteria at birth later associated with BPD development, *Ureaplasma* has been associated with an increased risk for developing BPD (27, 28). Chorioamnionitis has been cited as an independent risk factor for BPD for a very long time and we reported that preterm infants born to mothers with chorioamnionitis start off with decreased *Lactobacilli* in their airways at birth. Over time, a decrease in *Acinetobacter* abundance and increase in *Staphylococcus* and *Klebsiella* abundance has been reported in infants that develop BPD (20). However, these investigations of bacterial abundance and BPD susceptibility do not detail the mechanism for altered pulmonary development.

Recently, we conducted another study to using a software-based method to predict the metagenome of the tracheal aspirate microbiome from 16S rRNA sequencing data in extremely preterm infants (born at ≤ 28 weeks' gestation) and identified functional host ortholog genes that were differentially abundant in infants developing BPD compared to infants that did not develop BPD (33). We also identified metabolites and metabolic pathways that were differentially enriched in these samples by use of untargeted mass spectrometry and mummichog. The airway metabolome of infants developing BPD was enriched for metabolites involved in fatty acid activation and androgen and estrogen biosynthesis compared with infants that did not develop BPD. These findings suggest that in extremely preterm infants, the early airway microbiome may alter the metabolome, thereby modifying the risk of BPD. While the etiology of differential enrichment of sex steroid metabolic pathways remains unclear, previous studies have suggested a role for sexual dimorphism in BPD risk (33).

## From ‘Biome to BPD: Mechanisms of Consideration

Proposed mechanisms by which the airway microbiome results in BPD development include immune system priming, oxidative stress, and metabolic dysregulation. The immunomodulatory role of the airway microbiome in human disease has been well-described (34–36). Previous investigations have reported a decrease in inflammatory cytokines (e.g., tumor necrosis factor-α) in peripheral blood mononuclear cells following exposure to *Lactobacillus*-derived factors (37). Additionally, in *in vitro* studies of LPS-induced lung injury, inflammatory cytokines derived from alveolar macrophages were reduced upon exposure to *Lactobacillus-*derived factors (38). *In vivo* studies also reported less recruitment of inflammatory cells to the lung tissue with *Lactobacillus* administration (38). Instillation of *Lactobacilli* into adult wild-type mice lungs preserves alveolar architecture compared to germ-free mice controls (39). Nevertheless, neonatal germ-free mice have relatively protected alveolar structure in response to noxious stimuli such as hyperoxia (40). The probiotic use of *Lactobacilli* has already been demonstrated in the setting of enteropathogenic colonization and viral infection (41).

We have reported differences in exosome signatures between infants who do and do not develop BPD. Exosomes are vesicles produced by numerous cells and house molecular content, and may participate in cellular communication and pathogenic pathways, and may therefore provide clinical utility as a biomarker for disease. In a large prospective study in extremely preterm infants (born at <28 weeks' gestation), exosomes were longitudinally collected from tracheal aspirates and compared between infants that did and did not develop severe BPD (42). Infants developing severe BPD had 40 different micro RNA signatures compared to non-BPD infants. Upon analyzing these signatures in a validation cohort, low miR 876-3p expression had a sensitivity of 91.7% for severe BPD prediction (42). As previously discussed, Proteobacteria are more abundant in infants that develop severe BPD and using an *in vivo*, hyperoxia-based BPD mouse model, we compared the alveolar architecture of hyperoxia exposed mice to hyperoxia exposed mice also exposed to Proteobacterial LPS. Mice exposed to hyperoxia and *Proteobacterial* LPS had the most significant reduction in miR 876-3p expression supporting the role of miR 876-3p as a potential biomarker for *Proteobacterial-*induced BPD development and/or a therapeutic target (42). As miR 876-3p was found to be a protective miRNA in BPD pathogenesis, gain of function of miR 876-3p could be attempted in therapeutic studies of chronic lung injury. Additionally, further studies utilizing miR 876-3p to validate BPD prediction at other sites, to monitor therapeutic response to therapy, and/or predict other outcomes in extremely preterm infants (e.g., long term pulmonary morbidity) are needed. Interestingly based on bioinformatic platforms, one of the top predicted targets of miR 876-3p is androgen receptor (AR). We speculate that further study of miR 876-3p and its target AR has the potential to delineate the sex predilection seen in BPD.

Given that a dysbiotic airway microbiome has been associated with BPD development, we have recently investigated whether germ-free mice exhibit a BPD phenotype after exposure to hyperoxia (40). In these studies, pulmonary development was similar between germ-free and non-germ-free (NGF) mice under normoxic conditions. In a hyperoxic environment, both NGF and germ-free mice exhibited evidence of alveolar maldevelopment; however, more alveolarization, decreased cytokine levels, and improved lung function were observed in germ-free mice compared to NGF mice (40). These experiments suggest that while the airway microbiome may not be a necessary exposure for lung maldevelopment, it may have an additive or synergistic role.

## Probiotics: A Plausible Therapeutic Option for BPD?

Several randomized trials have evaluated the effect of enteral probiotics and lung disease. In adults, probiotic exposure reduced ventilator-associated pneumonia compared to placebo in mechanically ventilated patients and reduced pulmonary exacerbations in patients with cystic fibrosis (43, 44). In a meta-analysis of probiotics in the context of allergic rhinitis, *Lactobacillus*-based probiotics were the probiotic used in the majority of studies and 22 randomized trials were identified. Probiotic-exposed patients had a reduction in symptom scores, an improvement in quality of life scores, and improved immunologic parameters compared to placebo (45). A randomized controlled trial of probiotics in children with asthma reported a reduction in asthma severity in probiotic exposed children compared to placebo and *Lactobacillus*-based probiotics may also reduce nosocomial infections in children (44, 46). Previous studies using enteral probiotics to reduce either necrotizing enterocolitis (NEC), a devastating gastrointestinal disease of prematurity, or late onset sepsis have not demonstrated a reduction in BPD (47). However, the influence of enteral probiotics on the airway microbiome has not been evaluated and randomized trials of aerosolized probiotics in preterm infants have never been conducted. Future studies are warranted to specifically target the lungs in animal models of respiratory diseases such as BPD, CF, and COPD. There may be a potential role of inhaled respiratory probiotics in the years to come.

## Future Investigation

Although bacteria such as *Lactobacilli* may be lung protective, there have been no studies demonstrating this protective role in animal models of neonatal chronic lung disease. Once the benefits of reversing the respiratory dysbiosis by using respiratory probiotics are established in small- and large-animal models, randomized controlled trials in extremely preterm infants would be needed. Despite numerous studies of associations between the microbiome and BPD development, further investigation into how a dysbiotic microbiome mechanistically predisposes infants to BPD development is also warranted. As the relative abundance of microbiota results from various environmental pressures including nutrient availability, temperature, and oxygen, as well as other determinants of temporospatial patterns including rate of reproduction, bacterial elimination, and immigration (30), it is unclear how such variables influence and may explain differences in the airway microbiome between BPD-resistant and -susceptible infants. Moreover, it remains unclear as to whether the microbiome causes or is associated with BPD development. As the origin of the airway microbiome remains uncertain and may precede birth, microbial targets upstream of airway colonization have not yet been identified.

## Author Contributions

All authors drafted, reviewed, edited, and finalized the manuscripts.

### Conflict of Interest

The authors declare that the research was conducted in the absence of any commercial or financial relationships that could be construed as a potential conflict of interest.
